# Análisis epidemiológico de la sífilis congénita en el departamento de Antioquia, Colombia, 2021 y 2022

**DOI:** 10.7705/biomedica.8040

**Published:** 2026-06-01

**Authors:** Natalia Arango-Serna, Natalia Carmona-Vargas, Alejandro D faz-Díaz, Carlos Andrés Vargas-Alzate

**Affiliations:** 1 Grupo de Investigación en Neurociencias y Envejecimiento, Facultad Ciencias de la Salud, Corporación Universitaria Remington, Medellín, Colombia Corporación Universitaria Remington Grupo de Investigación en Neurociencias y Envejecimiento Facultad Ciencias de la Salud Corporación Universitaria Remington Medellín Colombia; 2 Departamento de Pediatría y Enfermedades Infecciosas, Hospital General de Medellín, Colombia Departamento de Pediatría y Enfermedades Infecciosas Hospital General de Medellín Colombia; 3 Departamento de Pediatría y Enfermedades Infecciosas, Hospital Pablo Tobón Uribe, Medellín, Colombia Departamento de Pediatría y Enfermedades Infecciosas Hospital Pablo Tobón Uribe Medellín Colombia; 4 Departamento de Pediatría y Enfermedades Infecciosas, Hospital Infantil Concejo de Medellín, Medellín, Colombia Departamento de Pediatría y Enfermedades Infecciosas Hospital Infantil Concejo de Medellín Medellín Colombia

**Keywords:** sífilis congénita, vigilancia epidemiológica, edad gestacional, transmisión vertical de enfermedad infecciosa, salud materno-infantil, determinantes sociales de la salud, Syphilis, congenital, epidemiological monitoring, gestational age, infectious disease transmission, vertical, maternal and child health, social determinants of health

## Abstract

**Introducción.:**

La sífilis congénita es una enfermedad prevenible de alta morbimortalidad, cuya persistencia refleja fallas en el sistema de salud.

**Objetivo.:**

Caracterizar los reportes de sífilis congénita en el departamento de Antioquia en el 2021 y en el 2022 y su relación con la edad gestacional al momento del parto y del diagnóstico.

**Materiales y métodos.:**

Se llevó a cabo un estudio descriptivo, retrospectivo, de corte transversal. Se analizaron todos los casos de sífilis congénita reportados en Antioquia en el 2021 y en el 2022 al Instituto Nacional de Salud. Se describieron las variables sociodemográficas, clínicas y de atención incluidas en la ficha de vigilancia epidemiológica. Se calcularon las frecuencias, las medidas de resumen y las pruebas estadísticas para explorar las asociaciones.

**Resultados.:**

Se reportaron 375 casos de sífilis congénita, con incidencias de 2,9 y 2,3 por cada 1000 nacidos vivos en el 2021 y el 2022, respectivamente. El 54,4 % (204/375) de las madres era de bajo nivel socioeconómico y el 86,7 % (325/375) de los diagnósticos se hizo en el tercer trimestre. El 46,4 % (174/375) de las madres no tuvo control prenatal y el 17,6 % (66/375) no recibió penicilina antes del parto. La edad gestacional en el momento del parto se asoció significativamente con la condición final del neonato (p < 0,001), el control prenatal (p < 0,001), el tratamiento (p < 0,05) y los resultados serológicos de madre y neonato (p < 0,05).

**Conclusiones.:**

La sífilis congénita persiste como problema de salud pública en Antioquia, reflejo de deficiencias en el diagnóstico oportuno y tratamiento integral, además de la influencia de factores determinantes sociales como el bajo nivel socioeconómico. Por ende, se requiere fortalecer el primer nivel de atención, garantizar un tamizaje prenatal temprano e implementar estrategias diferenciadas por territorio que permitan reducir la transmisión vertical y avanzar hacia la eliminación de la enfermedad.

## Abstract (en)

**Introduction.:**

Congenital syphilis is a preventable disease with high morbidity and mortality, whose persistence reflects failures within the healthcare system.

**Objective.:**

To characterize reports of congenital syphilis in Antioquia in the years 2021 and 2022 and to analyze their relationship with gestational age at the time of birth and diagnosis.

**Materials and methods.:**

A descriptive, retrospective, cross-sectional study was conducted. All reported cases of congenital syphilis in Antioquia during 2021 and 2022 were analyzed using data from the *Instituto Nacional de Salud.* Sociodemographic, clinical, and healthcare-related variables from the epidemiological surveillance form were described. Frequencies, summary measures and statistical tests were applied to explore associations.

**Results.:**

A total of 375 cases of congenital syphilis were reported, with incidences of 2.9 and 2.3 per 1000 live births in 2021 and 2022, respectively. A total of 54.4% (204/375) of the mothers were of low socioeconomic status, and 86.7% (325/375) of diagnoses occurred in the third trimester. Additionally, 46.4% (174/375) of mothers did not receive prenatal care, and 17.6% (66/375) did not receive penicillin before delivery. Gestational age at delivery was significantly associated with the neonate's final condition (p < 0.001), prenatal care (p < 0.001), treatment (p < 0.05), and maternal-infant serological outcomes (p < 0.05).

**Conclusions.:**

Congenital syphilis remains a public health concern in Antioquia, reflecting deficiencies in timely diagnosis and comprehensive treatment, as well as the influence of social determinants such as low socioeconomic status. Consequently, it is essential to strengthen primary healthcare services, ensure early and continuous prenatal screening, and implement territory-specific strategies aimed at reducing vertical transmission and advancing toward the elimination of the disease.

La sífilis congénita es una enfermedad prevenible con alta morbimortalidad que, a pesar de los avances en la detección y en el tratamiento de la sífilis gestacional, continúa siendo un problema de salud pública a nivel mundial ^1^. La sífilis congénita se origina por la transmisión vertical de *Treponema pallidum* de la madre al feto durante la gestación o en el parto, lo que puede derivar en complicaciones graves como parto prematuro, bajo peso al nacer, muerte perinatal y alteraciones neurológicas y auditivas del recién nacido ^2^.

Según estimaciones de la Organización Mundial de la Salud (OMS), en el 2022 se registraron cerca de 8 millones de nuevos casos de sífilis en adultos entre los 15 y los 49 años, y de manera independiente, aproximadamente, 700 000 casos de sífilis congénita, 150 000 muertes fetales tempranas y prenatales, 70 000 muertes neonatales, 55 000 partos prematuros o de neonatos con bajo peso al nacer y 115 000 casos con diagnóstico clínico de sífilis congénita del lactante ^1^^,^^3^. En la región de las Américas, se estima que hubo alrededor de 3,36 millones de nuevos casos de sífilis, 183 000 mujeres embarazadas con sífilis gestacional y 68 000 casos de sífilis congénita ^3^.

En Colombia, en la semana epidemiológica 35 del 2022, se reportó una prevalencia nacional de 15,7 casos de sífilis gestacional por 1000 nacidos vivos más mortinatos y una incidencia de 2,9 casos de sífilis congénita por cada 1000 nacidos vivos más mortinatos ^4^. Las entidades territoriales de Arauca, Buenaventura, Chocó, Guainía y Vichada presentaron las prevalencias de más altas, con cifras entre 27,3 y 43,2 casos por 1000 nacidos vivos más mortinatos ^4^.

En cuanto a la sífilis congénita, la incidencia para el 2023 fue de 2,4 casos por cada 1000 nacidos vivos más mortinatos, cifra que superó la meta de 0,5 casos de reducción establecida para ese mismo periodo ^5^. Respecto a las entidades territoriales, el 47,6 % superó la cifra a nivel nacional y las más altas se reportaron en los departamentos de Casanare, Arauca y Norte de Santander ^5^.

Antioquia es el segundo departamento con mayor población en Colombia y se ha identificado como una de las regiones con gran cantidad de casos de sífilis congénita. Según las fuentes oficiales, en Antioquia se han reportado incidencias de 2,5 y 1,4 casos de sífilis congénita por cada 1000 nacidos vivos más mortinatos en los años 2021 y 2022, respectivamente ^6^^,^^7^. Aunque desde el 2003 a nivel nacional es obligatorio notificar esta enfermedad al Sistema de Vigilancia en Salud Pública (SIVIGILA), persisten dificultades para la detección temprana de los casos y la iniciación oportuna de los tratamientos ^8^.

En este contexto, el análisis de la edad gestacional adquiere una relevancia importante, dado que permite conocer no solo el momento en que ocurre el diagnóstico materno, sino también el grado de desarrollo fetal en el momento del parto, ambos factores decisivos en el pronóstico del recién nacido. La edad gestacional puede actuar como un marcador crítico que puede revelar fallas en el tamizaje prenatal, brechas en el acceso al tratamiento oportuno y retrasos en la atención dentro del sistema de salud ^9^^,^^10^.

Teniendo en cuenta que a nivel local no se han realizado estudios que evalúen la relación entre los casos de sífilis congénita y la edad gestacional, se hace necesario investigarla a profundidad, pues su análisis no solo contribuye a una mejor comprensión de los perfiles clínico-epidemiológicos, sino que, además, orienta la formulación de estrategias focalizadas de prevención, atención y control en contextos territoriales específicos.

Por lo descrito anteriormente, el presente estudio tuvo como propósito caracterizar los casos de sífilis congénita reportados en el departamento de Antioquia en los años 2021 y 2022 y analizar su relación con la edad gestacional en el momento del parto y del diagnóstico. Esta información permitirá identificar patrones epidemiológicos relevantes y orientar el diseño de estrategias de prevención y control más eficaces en la región.

## Materiales y métodos

Se realizó un estudio descriptivo, retrospectivo y de corte transversal.

Se incluyeron los registros de los pacientes reportados con sífilis congénita en el departamento de Antioquia durante los años 2021 y 2022, mediante las fichas epidemiológicas del SIVIGILA, entidad que hace parte del Ministerio de Salud y Protección Social. Se excluyeron del estudio los casos reportados con fichas epidemiológicas incompletas o mal diligenciadas y las fichas duplicadas que no fueron por reinfección. Al incluir los reportes, no se calculó un tamaño de muestra ni se aplicaron técnicas de muestreo.

### Recolección de la información

Se utilizó como fuente de información el reporte de datos consolidado de las fichas de vigilancia epidemiológica para la sífilis congénita (código 740 del Instituto Nacional de Salud) con la última versión disponible del 8 de junio del 2022. Mediante un convenio institucional, se accedió al listado de todos los casos notificados y confirmados como casos positivos por parte de la Dirección Seccional de Antioquia según el protocolo de vigilancia establecido por el SIVIGILA ^11^.

Luego, se llevó a cabo la validación y depuración de las siguientes variables: edad, nacionalidad, departamento y municipio de procedencia, tipo de régimen en salud, pertenencia étnica, estrato, grupo poblacional al que pertenece, hospitalizado, condición final, diagnóstico materno (condición en el momento del diagnóstico, control prenatal del embarazo, edad gestacional en el momento del diagnóstico, prueba treponémica y no treponémica con su resultado), tratamiento materno (penicilina benzatínica - número de dosis recibidas antes del parto, fecha de aplicación de la primera dosis en la mujer gestante, tratamiento de contactos) y evaluación del riesgo de sífilis congénita (resultado de la gestación, número de productos al nacimiento, que se categorizaran en 1, 2 o mayor de 3 productos, edad gestacional en el momento del parto, resultado de la serología de la madre en el momento del parto, resultado de la serología del recién nacido).

Las variables de edad gestacional en el momento del diagnóstico y edad gestacional en el momento del parto, que originalmente fueron cuantitativas, se categorizaron según lo observado en la literatura y la experiencia profesional de los investigadores en el ámbito clínico, de la siguiente manera: edad gestacional en el momento del parto en recién nacido prematuro (de 24 a 34 semanas), recién nacido a término (37 a 42 semanas) y postérmino (mayor a 42 semanas), y edad gestacional en el momento del diagnóstico en primer trimestre (de 1 a 3 meses), segundo trimestre (de 4 a 6 meses) y tercer trimestre (de 7 a 9 meses) ^11^^,^^12^.

### Análisis de la información

Se calcularon frecuencias (absolutas y relativas) para las variables cualitativas y medianas con su rango intercuartílico para las cuantitativas debido al incumplimiento del supuesto de normalidad según la prueba de Shapiro Wilk. La incidencia de sífilis congénita se calculó mediante la fracción del número de casos nuevos reportados por sífilis congénita en el periodo de estudio sobre el total de nacidos vivos (incluido mortinatos) notificados por el Departamento Administrativo Nacional de Estadísticas (DANE) en el mismo periodo de tiempo y se utilizó 1000 como constante ^11^^,^^13^.

Para la exploración de las asociaciones estadísticas entre las características sociodemográficas y las clínicas con las edades gestacionales categorizadas, se utilizaron pruebas como: X^2^ de Pearson, X^2^ de tendencia lineal, prueba exacta de Fisher, U de Mann-Whitney y Kruskal Wallis. Como análisis *post hoc,* se hicieron comparaciones múltiples por columna para detallar las diferencias estadísticas entre las variables categóricas y la corrección de Bonferroni para las continuas. Se utilizó un nivel de significancia estadística de p < 0,05 y los datos se procesaron en el *software* estadístico Jamovi 2.6.23 (versión libre) y *Statistical Package for the Social Sciences™,* SPSS, versión 22.

### Consideraciones éticas

El estudio fue aprobado por el Comité de Bioética de la Corporación Universitaria Remington de Medellín con número de acta 072023, y fue clasificado como «sin riesgo» según la Resolución 8430 de 1993 del Ministerio de Salud y Protección Social; además, se ajustó a las normas correspondientes para la investigación con información sensible.

## Resultados

**Figura 1:**
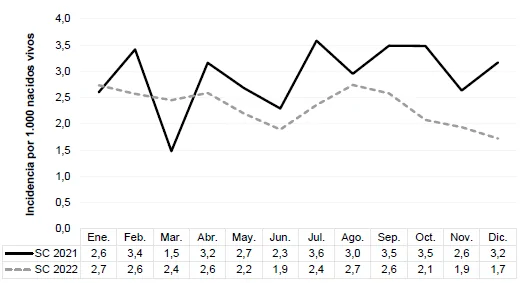
Incidencia de sífilis congénita en el departamento de Antioquia, 2021 y 2022

Se reportaron 375 casos de neonatos con sífilis congénita en el departamento de Antioquia en el 2021 (56,8 %) y en el 2022 (43,2 %), con incidencias de 2,9 y 2,3 casos por cada 1000 nacidos vivos, respectivamente. El 2021 fue el de mayor variabilidad en la incidencia mensual, mientras que las incidencias del 2022 no superaron los 2,7 casos por cada 1000 nacidos vivos, con una disminución constante en los últimos meses (figura 1).

SC: sífilis congénita

**Figura 2:**
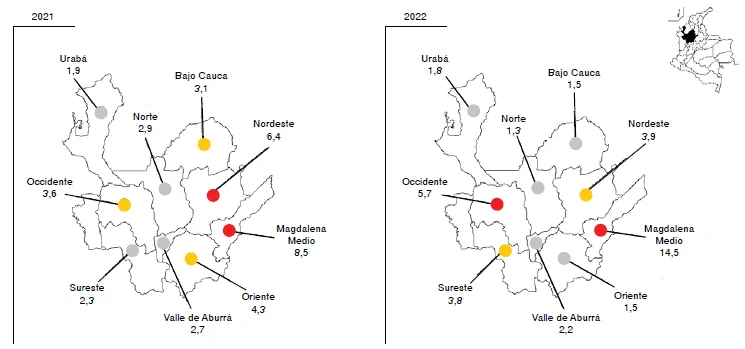
Incidencia de sífilis congénita por subregión de Antioquia, 2021 y 2022

Respecto a las subregiones del departamento, el Magdalena medio fue el de mayores incidencias, pasando de 8,5 (2021) a 14,5 (2022) casos por cada 1000 nacidos vivos y superando en el 154 % a la segunda región más frecuente en el 2022 (occidente) (figura 2).

En el cuadro 1 se presentan las características sociodemográficas de los pacientes reportados con sífilis congénita. Se encontró que el 54,4 % (n = 204) de los menores reportados con esta enfermedad era de sexo masculino; la mitad tenía 4 días o más de nacidos; ocho de cada diez casos requirieron hospitalización y el 7,5 % (n = 28) tuvo como condición final la muerte (25 de los 28 fueron mortinatos).

**Cuadro 1:** Características sociodemográficas de los casos reportados con sífilis congénita, Antioquia, 2021 y 2022 (N= 375)

			n (%)
Características de los neonatos	Sexo	Mujer	170 (45,3)
		Hombre	204 (54,4)
	Edad (días) - Mediana (RI)*	Sin dato	1 (0,3)
		4 (2 a 8)	
	Nacionalidad	Colombia	371 (98,9)
		Otros países	4 (1,1)
	Hospitalizado	Sí	316 (84,3)
		No	59 (15,7)
	Condición final	Vivo	346 (92,3)
		Muerto	28 (7,5)
		Sin dato	1 (0,3)
Características de las madres	Ubicación de la vivienda	Cabecera municipal	293 (78,1)
		Centro poblado	28 (7,5)
		Rural disperso	54 (14,4)
	Tipo de seguridad social	Subsidiado	154 (41,1)
		Contributivo	74 (19,7)
		Excepción	3 (0,8)
		No asegurado	58 (15,5)
		Indeterminado	86 (22,9)
	Pertenencia étnica	Indígena	11 (2,9)
		Negro, mulato, afrocolombiano	7 (1,9)
		Ninguna	357 (95,2)
	Estrato socioeconómico	1	103 (27,5)
		2	101 (26,9)
		3	83 (22,1)
		Sin dato	88 (23,5)
	Número de recién nacidos	Uno	366 (97,6)
		Dos	9 (2,4)
	Resultado de la gestación	Recién nacido vivo	350 (93,3)
		Mortinato	25 (6,7)
	Desplazados	Sí	1 (0,3)
		No	365 (97,3)
		Sin dato	9 (2,4)
	Migrantes	Sí	35 (9,3)
		No	331 (88,3)
		Sin dato	9 (2,4)
	Habitantes de calle	Sí	2 (0,5)
		No	364 (97,1)
		Sin dato	9 (2,4)

RI: rango intercuartílico

* Mínimo = 0,03; máximo = 270

Con respecto a las madres, se encontró que vivían principalmente en las cabeceras municipales, cerca del 5 % pertenecía a alguna etnia y todas pertenecían al estrato socioeconómico medio o bajo. Además, se observó que el 9,9 % (n = 37) de las madres tenía alguna condición de vulnerabilidad, y la más frecuente fue la condición de migrante con el 9,3 % (n = 35).

Referente al tratamiento y seguimiento de las madres (cuadro 2), el 53,6 % (n = 201) tuvo control prenatal del embarazo; una quinta parte de ellas (n = 73) tuvo un diagnóstico de reinfección; el 79,2 % (n = 297) recibió penicilina benzatínica antes del parto y solamente el 41,3 % (n = 155) de los contactos tuvo tratamiento. En la serología materna en el momento del parto, los títulos de anticuerpos medidos mediante pruebas no treponémicas que prevalecieron estuvieron entre 1:4 a 1:64 diluciones (63,2 %; n = 237). Por su parte, en la serología de los recién nacidos, el 32,5 % (n = 122) presentó títulos iguales o menores de 1:2 diluciones y el 16,5 % (n = 62) tuvo resultados no reactivos.

**Cuadro 2:** Tratamiento y seguimiento a las madres y menores con sífilis congénita, Antioquia, 2021 y 2022 (N = 375)

Tratamiento y seguimiento		n (%)
Control prenatal en embarazo actual	Sí	201 (53,6)
	No	174 (46,4)
Diagnóstico del embarazo actual	Primera vez	302 (80,5)
	Reinfección	73 (19,5)
Prueba treponémica	Sí	363 (96,8)
	Sin dato	12 (3,2)
Tipo de prueba treponémica	Prueba rápida	328 (87,5)
	Otra	35 (9,3)
	Sin dato	12 (3,2)
Resultado de la prueba treponémica	Positiva	363 (96,8)
	Sin dato	12 (3,2)
Penicilina benzatínica antes del parto (dosis)	0	66 (17,6)
	1	212 (56,5)
	2	30 (8)
	3	55 (14,7)
	Sin dato	12 (3,2)
Tratamiento de contactos	Sí	155 (41,3)
	No	220 (58,7)
Serología de la madre en el momento del parto (VDRL o RPR) (diluciones)	≤ 1:2	120 (32)
	1:4	39 (10,4)
	1:8	43 (11,5)
	1:16	59 (15,7)
	1:32	53 (14,1)
	1:64	43 (11,5)
	1:128	11 (2,9)
	1:256	6 (1,6)
	1:512	1 (0,3)
Serología del recién nacido (VDRL o RPR) (diluciones)	≤ 1:2	122 (32,5)
	1:4	50 (13,3)
	1:8	39 (10,4)
	1:16	29 (7,7)
	1:32	22 (5,9)
	1:64	16 (4,3)
	1:128	4 (1,1)
	1:256	3 (0,8)
	1:512	2 (0,5)
	1:1024	1 (0,3)
	No reactiva	62 (16,5)

RPR: *Rapid Plasma Reagin;* VDRL: *Venereal Disease Research Laboratory*

**Figura 3:**
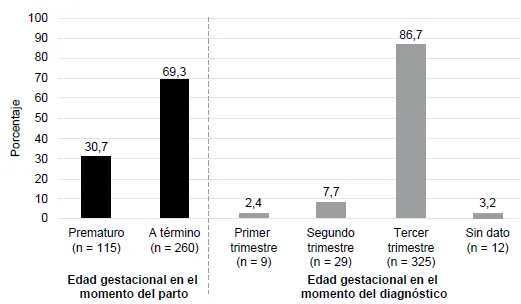
Distribución de los casos de sífilis congénita según la edad gestacional al momento del parto y al momento del diagnóstico, Antioquia, 2021 y 2022 (N = 375)

En relación con la edad gestacional al momento del parto, el 69,3 % (n = 260) de los casos de sífilis congénita fue reportado a término, mientras que, según la edad gestacional en el momento del diagnóstico, fue mayor la frecuencia de casos reportados en el tercer trimestre del embarazo (86,7 %; n = 325) (figura 3).

Finalmente, en el cuadro 3 se observa que la edad gestacional en el momento del parto presentó asociación estadística con la condición final del neonato (p < 0,001), el número de productos al nacimiento (p < 0,02), el control prenatal del embarazo (p < 0,001), el número de dosis recibidas de penicilina benzatínica (p < 0,001), el tratamiento de los contactos (p < 0,03), y los resultados de serología de la madre (p < 0,05) y del recién nacido (p < 0,001).

**Cuadro 3:** Casos reportados con sífilis congénita según la edad gestacional al momento del parto y del diagnóstico, Antioquia, 2021 y 2022 (N = 375)

			Edad gestacional						
			Parto		p	Diagnóstico*			p
			Prematuro (n = 115) n (%)	A término (n = 260) n (%)		Primer trimestre (n = 9) n (%)	Segundo trimestre (n = 29) n (%)	Tercer trimestre (n = 325) n (%)	
Características de los neonatos	Sexoa	Mujer	46 (40,0)	124 (47,7)	0,136	6 (66,7)	15 (51,7)	145 (44,6)	0,689
		Hombre	68 (59,1)	136 (52,3)		3 (33,3)	14 (48,3)	179 (55,1)	
	Edad (días) mediana (RI)	Edad (días)	4 (2 a 10)	4 (2 a 8)	0,516	6 (5-10)	7 (2 a 13)	4 (2 a 8)	0,203
	Nacionalidad	Colombia	115 (100)	256 (98,5)	0,229	9 (100)	29 (100)	321 (98,8)	0,789
		Otros países	0 (0,0)	4 (1,5)		0 (0,0)	0 (0,0)	4 (1,2)	
	Hospitalizado	Sí	88 (76,5)	228 (87,7)	0,006**	7 (77,8)	20 (69,0)	280 (86,2)	0,042**
		No	27 (23,5)	32 (12,3)		2 (22,2)	9 (31,0)	45 (13,8)	
	Condición finala	Vivo	90 (78,3)	256 (98,5)	<0,001**	9 (100)	24 (82,8)	305 (93,8)	0,174
		Muerto	25 (21,7)	3 (1,2)		0 (0,0)	5 (17,2)	19 (5,8)	
Características de las madres	Ubicación de la vivienda	Cabecera municipal	97 (84,3)	196 (75,4)	0,054	8 (88,9)	18 (62,1)	257 (79,1)	0,045**
		Centro poblado	9 (7,8)	19 (7,3)		1 (11,1)	6 (20,7)	21 (6,5)	
		Rural disperso	9 (7,8)	45 (17,3)		0 (0,0)	5 (17,2)	47 (14,5)	
	Tipo de seguridad social	Subsidiado	57 (49,6)	97 (37,3)	0,173	4 (44,4)	15 (51,7)	128 (39,4)	0,557
		Contributivo	18 (15,7)	56 (21,5)		3 (33,3)	7 (24,1)	64 (19,7)	
		Desconocido	25 (21,7)	61 (23,5)		0 (0,0)	5 (17,2)	80 (24,6)	
		No Asegurado	15 (13,0)	43 (16,5)		2 (22,2)	2 (6,9)	50 (15,4)	
	Pertenencia étnica	Indígena	2 (1,7)	9 (3,5)	0,522	8 (88,9)	27 (93,1)	311 (95,7)	0,189
		Negro, mulato afrocolombiano	3 (2,6)	4 (1,5)		1 (11,1)	1 (3,4)	4 (1,2)	
	Estrato socioeconómicoa	1	30 (26,1)	73 (28,1)	0,725	2 (22,2)	8 (27,6)	88 (27,1)	0,866
		2	31 (27,0)	70 (26,9)		4 (44,4)	8 (27,6)	83 (25,5)	
		3	23 (20,0)	60 (23,1)		2 (22,2)	5 (17,2)	76 (23,4)	
	Número de productos al nacimiento	1	109 (94,8)	257 (98,8)	0,027***	9 (100)	29 (100)	316 (97,2)	0,583
		2	6 (5,2)	3 (1,2)		0 (0,0)	0 (0,0)	9 (2,8)	
**Características del seguimiento y tratamiento**									
Control prenatal en embarazo actual		Sí	43 (37,4)	158 (60,8)	<0,001**	7 (77,8)	19 (65,5)	168 (51,7)	0,120
		No	72 (62,6)	102 (39,2)		2 (22,2)	10 (34,5)	157 (48,3)	
Diagnóstico embarazo actual		Primera vez	96 (83,5)	206 (79,2)	0,338	6 (66,7)	21 (72,4)	266 (81,8)	0,260
		Reinfección	19 (16,5)	54 (20,8)		3 (33,3)	8 (27,6)	59 (18,2)	
Penicilina benzatínica - número de dosis recibidas antes del parto		0	18 (15,7)	48 (18,5)	0,001**	0 (0,0)	4 (13,8)	62 (19,1)	0,020**
		1	72 (62,6)	140 (53,8)		5 (55,6)	13 (44,8)	194 (59,7)	
		2	6 (5,2)	24 (9,2)		0 (0,0)	3 (10,3)	27 (8,3)	
		3	10 (8,7)	45 (17,3)		4 (44,4)	9 (31,0)	42 (12,9)	
Tratamiento de contactos		Sí	38 (33,0)	117 (45,0)	0,030**	6 (66,7)	5 (17,2)	139 (42,8)	0,008**
		No	77 (67,0)	143 (55,0)		3 (33,3)	24 (82,8)	186 (57,2)	
Serología de la madre en el momento del parto (VDRL o RPR)		≤ 1:2	24 (20,9)	96 (36,9)	0,042**	2 (22,2)	6 (20,7)	109 (33,5)	0,014**
		1:4	10 (8,7)	29 (11,2)		2 (22,2)	3 (10,3)	33 (10,2)	
		1:8	13 (11,3)	30 (11,5)		3 (33,3)	2 (6,9)	34 (10,5)	
		1:16	22 (19,1)	37 (14,2)		1 (11,1)	9 (31,0)	49 (15,1)	
		1:32	22 (19,1)	31 (11,9)		0 (0,0)	1 (3,4)	50 (15,4)	
		1:64	17 (14,8)	26 (10,0)		1 (11,1)	5 (17,2)	35 (10,8)	
		1:128	3 (2,6)	8 (3,1)		0 (0,0)	2 (6,9)	9 (2,8)	
		1:256	3 (2,6)	3 (1,2)		0 (0,0)	0 (0,0)	6 (1,8)	
		1:512	1 (0,9)	0 (0,0)		0 (0,0)	1 (3,4)	0 (0,0)	
Serología del recién nacido (VDRL o RPR)		≤ 1:2	26 (22,6)	96 (36,9)	<0,001**	4 (44,4)	7 (24,1)	107 (32,9)	0,715
		1:4	12 (10,4)	38 (14,6)		1 (11,1)	4 (13,8)	42 (12,9)	
		1:8	10 (8,7)	29 (11,2)		1 (11,1)	4 (13,8)	34 (10,5)	
		1:16	7 (6,1)	22 (8,5)		2 (22,2)	0 (0,0)	27 (8,3)	
		1:32	13 (11,3)	9 (3,5)		0 (0,0)	3 (10,3)	19 (5,8)	
		1:64	5 (4,3)	11 (4,2)		1 (11,1)	3 (10,3)	12 (3,7)	
		1:128	3 (2,6)	1 (0,4)		0 (0,0)	0 (0,0)	4 (1,2)	
		1:256	2 (1,7)	1 (0,4)		0 (0,0)	0 (0,0)	2 (0,6)	
		1:512	1 (0,9)	1 (0,4)		0 (0,0)	0 (0,0)	2 (0,6)	
		1:1024	0 (0,0)	1 (0,4)		0 (0,0)	0 (0,0)	1 (0,3)	
		No reactiva	13 (11,3)	49 (18,8)		0 (0,0)	4 (13,8)	58 (17,8)	

RI: rango intercuartílico; RPR: *Rapid Plasma Reagin*; VDRL: *Venereal Disease Research Laboratory*

* Doce registros no tenían registrada la edad al momento del diagnóstico.

** X^2^ de Pearson

*** Prueba exacta de Fisher

^a^ Sin dato

En cuanto a la edad gestacional al momento del diagnóstico, la categoría de tercer trimestre presentó cifras significativamente mayores a las reportadas en el primer y segundo trimestre en variables como la hospitalización del menor (p < 0,05), la ubicación de la vivienda (p < 0,05), el número de dosis recibidas de penicilina benzatínica (p < 0,030), el tratamiento de los contactos (p < 0,01), y los resultados de serología de la madre (p < 0,02).

## Discusión

Los resultados de este estudio confirman que la sífilis congénita sigue siendo un importante problema de salud pública en Antioquia, con incidencias que superan ampliamente las metas propuestas por la OMS para su eliminación. Aunque se observa una leve disminución entre el 2021 y el 2022, las cifras continúan evidenciando las deficiencias y los retos que existen en la prevención, el diagnóstico y el tratamiento oportuno de la sífilis en diferentes grupos poblacionales.

Uno de los hallazgos más preocupantes fue la alta proporción de diagnósticos maternos obtenidos en el tercer trimestre del embarazo (89,5 %); de estas madres, el 86 % de los neonatos requirió hospitalización y el 5,8 % murió. Esta identificación tardía limita las posibilidades de tratamiento efectivo y aumenta el riesgo de transmisión vertical, resultados que son similares a otros estudios de las Américas ^14^^-^^16^. En países como Argentina, Perú y México se encontró que en el 60 al 95 % de los casos de sífilis congénita, las madres no habían tenido controles prenatales o habían sido incompletos, situación que presentó una asociación significativa con desenlaces como el bajo peso al nacer y otras morbilidades ^6^^,^^17^^-^^20^.

Los estudios realizados en Barranquilla y en Bogotá demostraron que el inicio tardío del control prenatal aumentaba el riesgo de un parto prematuro y que factores como el bajo nivel educativo, la situación de migrante y el no estar afiliado al sistema de salud afectaban de forma negativa la posibilidad de tener un diagnóstico y un tratamiento oportunos ^21^^,^^22^. Este panorama da a conocer la necesidad de fortalecer los programas de tamizaje prenatal temprano, la vigilancia epidemiológica activa, las campañas educativas comunitarias y la atención en el primer nivel de atención, con el fin de garantizar un diagnóstico y tratamiento oportuno.

En cuanto al tratamiento, aunque más del 50 % de las madres con sífilis accedieron a controles prenatales, se encontró que aproximadamente una de cada cinco no recibió una dosis de penicilina benzatínica antes del parto y que el 19,5 % fueron casos de reinfección. Además, solo el 41,3 % de los contactos recibió tratamiento, proporción que fue menor entre las madres de neonatos prematuros. Estos resultados sugieren una falla estructural del sistema de salud, no solo de la disponibilidad del tratamiento, sino además del seguimiento clínico.

Algunos estudios realizados en Brasil y Perú han reportado que entre el 68,8 % y el 99,1 % de las madres de menores con sífilis congénita tenían un tratamiento inefectivo o no habían completado el tratamiento durante el embarazo, situación que está asociada de forma significativa a la ocurrencia de casos de sífilis congénita ^17^^,^^23^. Esta evidencia resalta que el diagnóstico por sí solo no es suficiente. El riesgo de transmisión vertical de *T. pallidum* persiste si no hay un tratamiento adecuado y completo para la madre y sus contactos. Por lo tanto, es imperativa la eliminación de las barreras estructurales, sociales y administrativas que dificultan el acceso y la finalización del manejo terapéutico de las mujeres gestantes ^24^^-^^26^.

La distribución territorial de los casos de sífilis congénita dio a conocer importantes diferencias. El Magdalena medio presentó un aumento relevante en la incidencia del 2021 y el 2022, mientras en el bajo Cauca y en el noreste disminuyeron hasta en el 50 % de un año a otro. Las diferencias territoriales encontradas en esta investigación resaltan la importancia de conocer la dinámica de las enfermedades desde enfoques diferenciados y según el contexto local.

En la región de las Américas, se han reportado frecuencias de sífilis congénita del 2 hasta del 69 %; en las poblaciones indígenas, del 54,2 % y en los países europeos entre el 6 y 76 % ^20^^,^^27^^-^^30^. La heterogeneidad de las cifras motiva la implementación de sistemas de monitoreo geoespaciales para focalizar intervenciones en zonas de alta vulnerabilidad y para fortalecer los equipos de salud pública en regiones con barreras estructurales persistentes con el fin de disminuir las brechas que limitan la implementación adecuada de los protocolos de atención.

Respecto a las condiciones sociodemográficas, en este estudio se evidenció una mayor frecuencia de los casos en mujeres de bajos estratos socioeconómicos, principalmente afiliadas al régimen subsidiado. La condición de vulnerabilidad más frecuente fue ser migrante. Estos resultados sugieren que los factores determinantes sociales de la salud siguen siendo relevantes en este tipo de enfermedades. En concordancia con otros trabajos, se ha documentado que las mujeres con menor escolaridad, sin pareja estable, que consumen drogas y en condición de migrantes, tienen mayores posibilidades de presentar transmisión vertical de la sífilis ^18^^,^^19^^,^^31^^-^^33^. En Colombia, otros estudios han establecido que, además, factores como la edad (menor de 21 años) y el lugar de residencia (zona rural y centro poblado) también influyen en la posibilidad de contraer sífilis congénita ^34^^,^^35^.

Estos datos resaltan la importancia de abordar los problemas de salud pública mediante la integración de dimensiones sociales, económicas y de marginación presentes en el entorno de las personas. Este enfoque permite formular estrategias focalizadas que contribuyen a superar las inequidades que perpetúan la transmisión vertical de la sífilis.

Un dato particularmente relevante fue la elevada proporción de títulos serológicos altos (≥ 1:32) de las madres en el momento del parto, situación que indica infección activa persistente durante la gestación, con mayor riesgo de alta carga infecciosa perinatal y mayores probabilidades de presencia de sífilis congénita sintomática. Varios estudios previos han demostrado que los títulos elevados del neonato están asociados con mayor gravedad clínica y con necesidad de un tratamiento más agresivo ^9^^,^^33^^,^^36^. Estos hallazgos subrayan la importancia del seguimiento serológico continuo durante el embarazo, así como del recién nacido, y sugieren la necesidad de incluir estos títulos como criterio de alerta temprana en los sistemas de vigilancia.

Este estudio presenta algunas limitaciones inherentes a su diseño transversal, las cuales impiden establecer relaciones de causalidad entre las variables analizadas, limitando los resultados a asociaciones descriptivas en un momento específico del tiempo. Además, se debe contemplar la existencia de posibles sesgos por subregistro, particularmente en las subregiones donde las barreras geográficas, estructurales y socioculturales pueden restringir el acceso a los servicios de atención.

En conclusión, la persistencia de la sífilis congénita en Antioquia pone en evidencia fallas estructurales en el acceso y la calidad del control prenatal, así como en el diagnóstico oportuno y el tratamiento integral de las mujeres gestantes. Este estudio resalta la necesidad de fortalecer el primer nivel de atención y garantizar un tamizaje prenatal y continuo, incorporando enfoques comunitarios que permitan superar las barreras culturales, económicas y sociales. Asimismo, se enfatiza la importancia de integrar la vigilancia epidemiológica con un análisis territorial diferenciado, que oriente las intervenciones adaptadas a las particularidades de cada región y sirva como modelo para avanzar hacia una eliminación de la sífilis congénita en Colombia.
